# Optical coherence tomography–defined radial artery calcification: frequency, phenotypes, and correlates in a retrospective transradial PCI cohort

**DOI:** 10.3389/fmed.2026.1945441

**Published:** 2026-10-27

**Authors:** Saiying He, Jia Zhou, Hao Liu, Haotian Wang, Zixuan Li, Jincheng Guo

**Affiliations:** 1 Department of Cardiology, Beijing Luhe Hospital, Capital Medical University, Beijing, China; 2 Department of Emergency Medicine, Beijing Luhe Hospital, Capital Medical University, Beijing, China

**Keywords:** coronary calcific nodule, optical coherence tomography, radial artery calcification, systemic calcific atherosclerosis, transradial access, vascular calcification

## Abstract

**Background and aims:**

The frequency, distribution, and optical coherence tomography (OCT) features of radial artery calcification (RAC) remain poorly defined. We assessed OCT-defined RAC and factors associated with patient-level RAC.

**Methods:**

This single-center retrospective observational study included 461 patients with analyzable radial artery OCT (RA-OCT) between January 2021 and August 2025. RAC was assessed at the patient, segment, and lesion levels. Clinical characteristics, radial artery plaque features, coronary OCT findings, and factors associated with patient-level RAC were evaluated.

**Results:**

Among 461 patients in the final analytic cohort, OCT-defined RAC was identified in 61 (13.2%). RAC involved the proximal, middle, and distal segments in 17/461 (3.7%), 25/461 (5.4%), and 52/461 (11.3%) patients, respectively. Among 214 calcified lesions, spotty calcification was the most common morphology (151/214, 70.6%). In exploratory patient-level analyses, all five radial artery plaque features were associated with RAC after multivariable adjustment, and all associations remained significant after Benjamini–Hochberg false discovery rate correction. In multivariable analysis, older age, diabetes mellitus, and coronary calcific nodule remained associated with RAC, whereas coronary calcium score was not statistically associated with RAC after adjustment.

**Conclusions:**

In this cohort, OCT-defined RAC was identified in 13.2% of patients, was more frequent in the distal segment, and showed heterogeneous lesion-level phenotypes. Older age, diabetes mellitus, and coronary calcific nodule were associated with RAC. Exploratory analyses also showed associations between RAC and multiple noncalcified radial artery plaque features.

## 1 Introduction

The radial artery is no longer merely an access site for cardiovascular procedures. It is a reusable vascular resource for coronary and neurovascular interventions, a conduit for coronary artery bypass grafting, and the arterial inflow for radiocephalic arteriovenous fistula creation (1–4). Preserving its structural integrity is therefore clinically important. Abnormalities such as small caliber, spasm, tortuosity, occlusion, prior instrumentation injury, and calcification may limit radial access, compromise conduit selection, and reduce future vessel usability (5–11).

Radial artery calcification (RAC) is a clinically relevant but incompletely defined abnormality. Early reports and subsequent studies have identified RAC in selected settings, including blood pressure measurement, preoperative assessment for radial artery grafting, chronic kidney disease, and distal radial access (12–15). These studies have linked RAC to clinical and vascular factors such as older age, diabetes mellitus, chronic kidney disease, mineral metabolism disorders, and coronary calcification or atherosclerotic burden (14, 15). However, most available data are based on ultrasound, radiography, computed tomography, or histology. These methods provide limited information on the *in vivo* longitudinal distribution, lesion morphology, calcification depth, and plaque context of RAC along the radial artery.

Optical coherence tomography (OCT) provides high-resolution *in vivo* imaging of the arterial wall and has been used to classify coronary calcification according to morphology, depth, arc, thickness, length, and nodular phenotype (16–18). Comparable OCT-based characterization of RAC is lacking. Whole-vessel radial artery OCT may define RAC beyond its presence or absence by assessing its spatial distribution, lesion-level morphology, calcification depth, and burden. In this study, we aimed to determine the observed frequency, spatial distribution, lesion-level OCT features, and clinical and coronary calcification correlates of OCT-defined RAC in patients undergoing transradial coronary procedures.

## 2 Materials and methods

### 2.1 Study design and population

Between January 2021 and August 2025, we conducted a single-center retrospective observational study of patients undergoing OCT-guided percutaneous coronary intervention (PCI) via radial access at Beijing Luhe Hospital, Capital Medical University. After completion of the coronary intervention, RA-OCT was attempted before the end of the same transradial procedure using the same OCT system and imaging catheter used for coronary OCT. RA-OCT was performed as part of the institutional imaging workflow, was not prompted by suspected RAC, and did not require a separate procedure or additional arterial access. Patients were excluded for inadequate image quality, left radial access, unsuccessful acquisition, incomplete whole-vessel coverage, previous right radial access, or current conventional right transradial access. Examinations acquired using non-OPTIS OCT systems were excluded to maintain uniform image-acquisition and quantitative-analysis protocols. No additional patients were excluded solely because of missing required clinical or imaging variables. The complete cohort derivation is shown in Figure 1.

**Figure 1:**
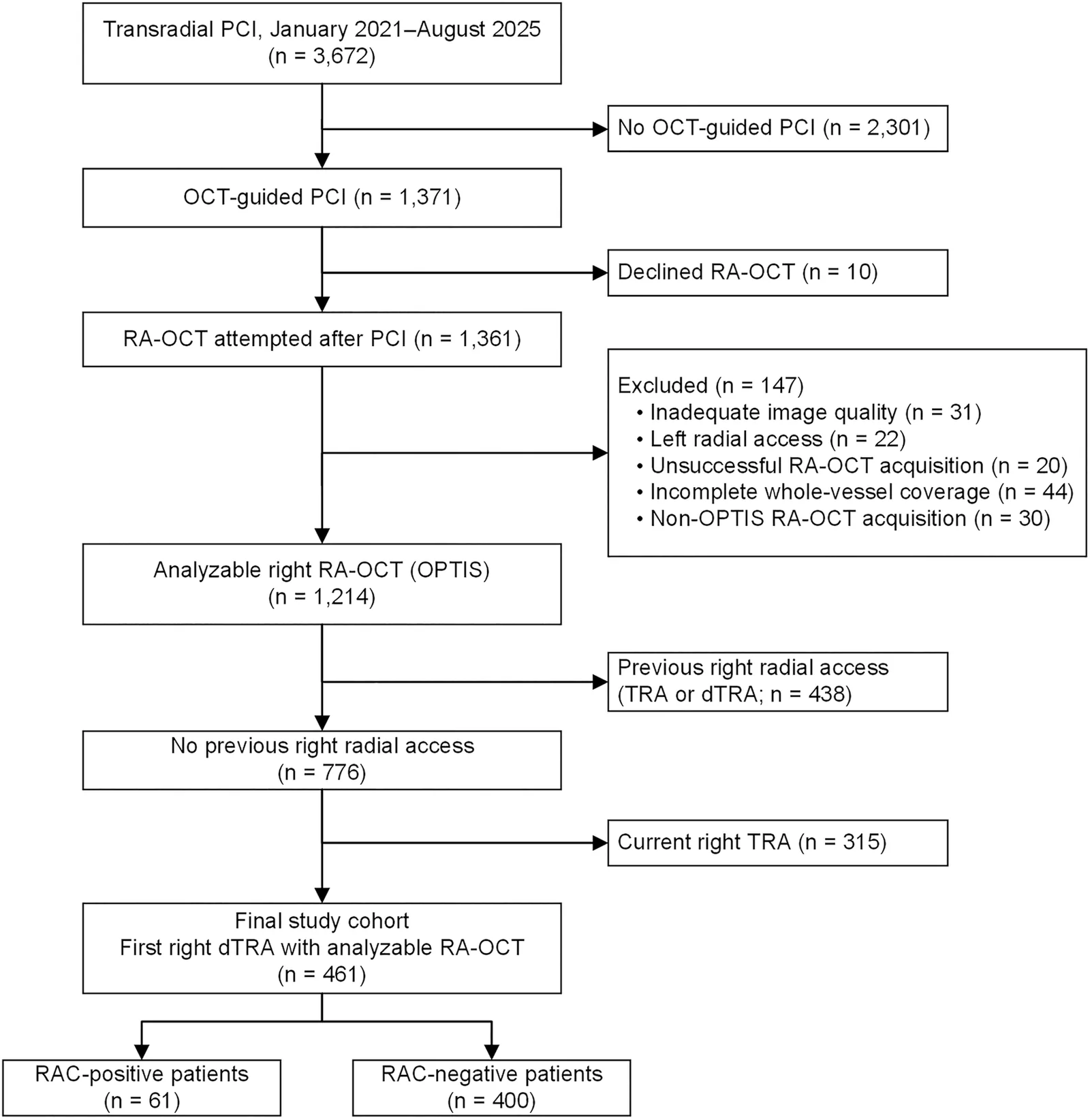
Study flowchart. Flow diagram showing the derivation of the final analytic cohort from 3,672 patients undergoing transradial PCI during the study period, including 10 patients who declined RA-OCT and subsequent exclusions based on imaging and access criteria. dTRA, distal transradial access; OCT, optical coherence tomography; PCI, percutaneous coronary intervention; RA, radial artery; RA-OCT, radial artery optical coherence tomography; TRA, conventional transradial access.

Patients were classified as RAC-positive or RAC-negative according to the presence or absence of OCT-defined RAC. The final analytic cohort included 61 RAC-positive and 400 RAC-negative patients. The study was approved by the Institutional Review Board of Beijing Luhe Hospital, Capital Medical University (approval no. 2021-LHKY-064-03).

### 2.2 Radial artery OCT acquisition

Radial artery OCT imaging was performed after completion of clinically indicated OCT-guided PCI and before the end of the same transradial procedure using a frequency-domain OCT system (OPTIS; Abbott Vascular, Santa Clara, CA, USA). A 0.014-inch guidewire was used to advance the OCT catheter to the radial artery ostium and was routinely withdrawn before image acquisition to improve circumferential visualization of the arterial wall, as previously described (17).

Motorized pullback was initiated from the radial artery ostium toward the distal radial artery during manual saline flushing through the radial sheath. Fluoroscopic landmarks, including a radiopaque ruler and an external metallic marker at the puncture site, were used for longitudinal localization and offline co-registration. OCT images were analyzed offline by trained analysts blinded to clinical data.

### 2.3 OCT analysis and definition of RAC

Using prespecified anatomical and pullback-based landmarks, the analyzable radial artery was divided into proximal, middle, and distal thirds. The radial artery ostium was used as the 0-mm reference point for longitudinal localization. The location of each RAC lesion was recorded as the distance from the radial artery ostium to the lesion midpoint on longitudinally co-registered OCT images. Mean radial artery diameter was calculated from analyzable cross-sections sampled at 5-mm intervals for the entire analyzable artery and for each segment. RAC was defined on OCT as a signal-poor or heterogeneous region with sharply delineated borders in the arterial wall, using established coronary OCT calcium criteria as a reference (18). RAC was assessed at the patient, segment, and lesion levels along the analyzable radial artery. Patient-level RAC was defined as at least one calcified lesion in any analyzable radial artery segment. Segment-level RAC was defined as calcification within the corresponding proximal, middle, or distal segment. A discrete RAC lesion was recorded when a calcified region had clearly distinguishable borders on serial OCT frames and was separated from adjacent calcification by intervening non-calcified vessel wall.

### 2.4 Morphological analysis of RAC lesions

RAC lesions were classified into three mutually exclusive morphologic patterns: spotty, sheet-like, and mixed-pattern calcification, using coronary OCT descriptors as a reference (18, 19). Spotty calcification was defined as a focal calcified deposit with length <4 mm and maximum arc <90°; sheet-like calcification was defined as a broad, plate-like, non-protruding calcified region; and mixed-pattern calcification was defined as the coexistence of spotty and sheet-like components within the same lesion. A calcific nodule was defined as a nodular calcific mass protruding into the lumen with an irregular luminal surface and was recorded separately as a non-mutually exclusive feature (Figure 2).

**Figure 2:**
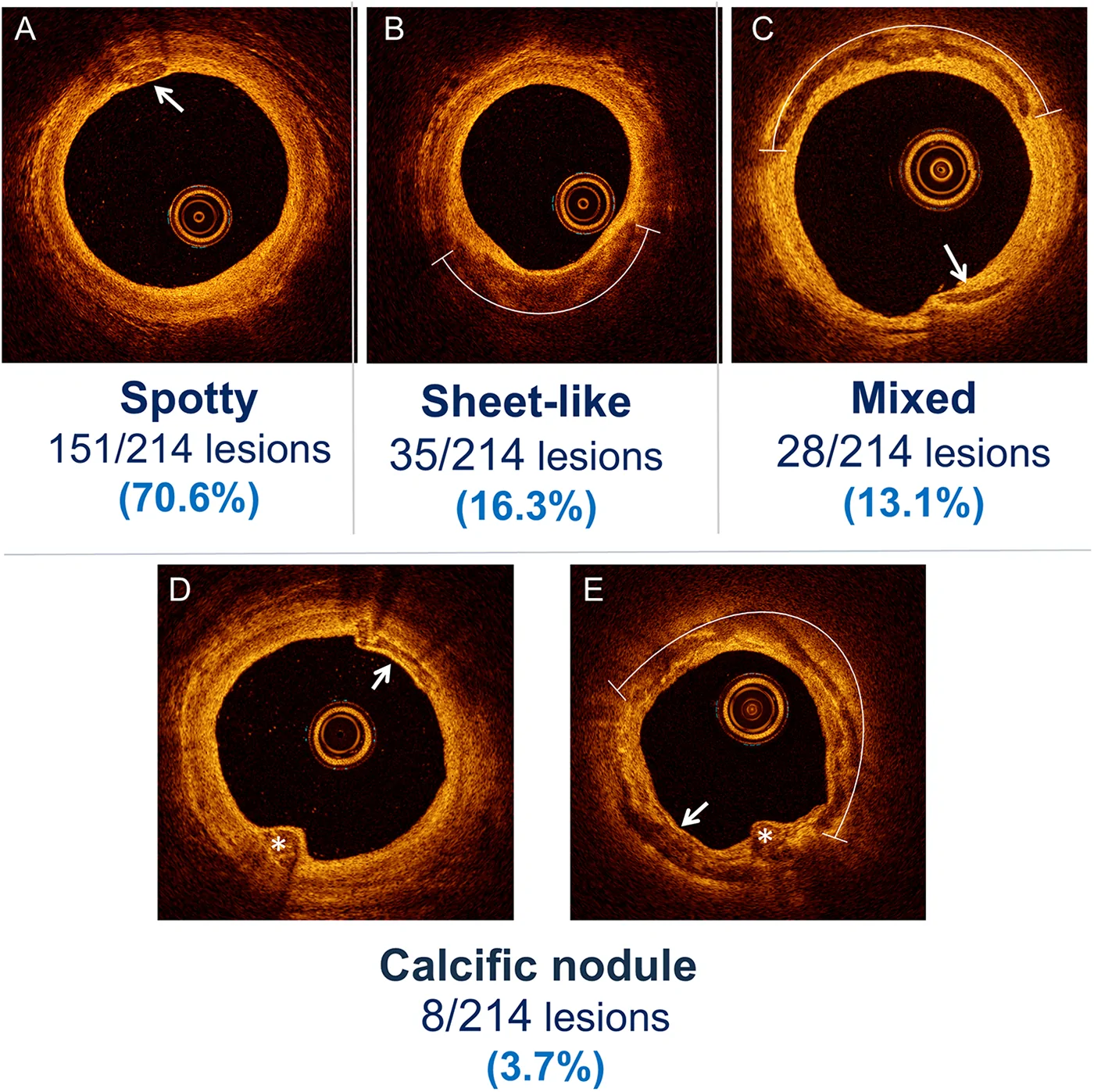
Representative OCT images and distribution of morphologic patterns of RAC lesions, with calcific nodules assessed separately. Panels **(A–C)** show the three mutually exclusive morphologic patterns—spotty, sheet-like, and mixed-pattern calcification—respectively. Panels **(D,E)** show representative calcific nodules, which were recorded separately as a non-mutually exclusive feature. Arrowheads indicate spotty calcification, brackets delineate sheet-like calcification, and asterisks indicate calcific nodules. Numerical frequencies are shown within the figure and are not repeated in the legend.

Depth-based classification used the minimum distance from the luminal surface to the luminal border of the calcified region. Using coronary OCT definitions, lesions with minimum depth ≤100 μm were classified as superficial and those with minimum depth >100 μm as deep (20). Lesions containing both superficial and deep components were classified as mixed-depth calcification (Figure 3). Outer-wall calcification was recorded separately during lesion-level assessment but was grouped with deep calcification for descriptive and graphical presentation. These categories were used as OCT descriptors rather than histological layer-specific diagnoses.

**Figure 3:**
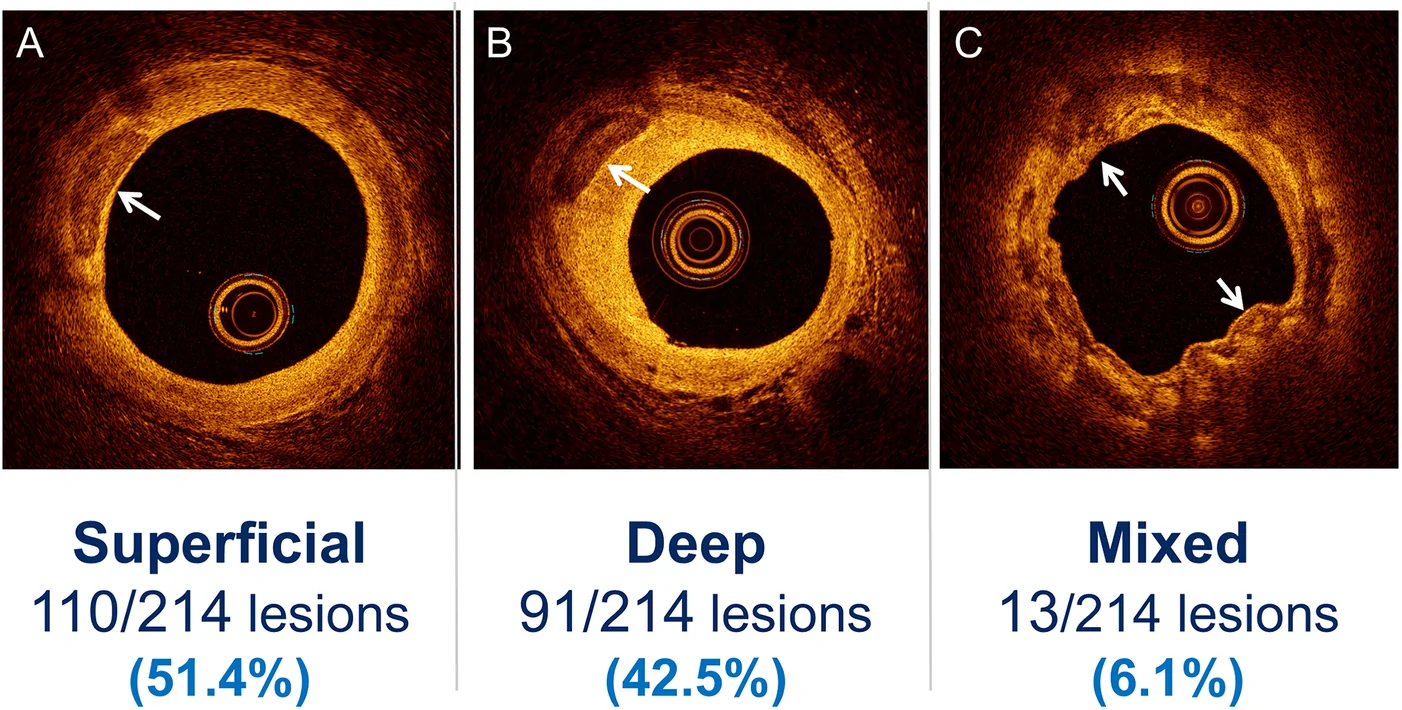
Representative OCT images and distribution of depth-based RAC patterns. Panels **(A–C)** show superficial, deep/outer-wall, and mixed-depth calcification, respectively. These categories are OCT descriptors and do not establish histological arterial-wall layers. OCT, optical coherence tomography; RAC, radial artery calcification.

Quantitative lesion-level measurements included lesion length, maximum calcium arc, maximum calcium thickness, and minimum calcium depth (Figure 4). Lesion length was calculated as the number of consecutive calcified frames multiplied by the inter-frame distance. Calcium arc was expressed in degrees; thickness was measured between the luminal and abluminal calcium borders; and depth was measured as the shortest distance from the luminal surface to the luminal calcium border. Calcification area was not analyzed because the complete abluminal contour was not consistently visible, precluding reproducible area measurement.

**Figure 4:**
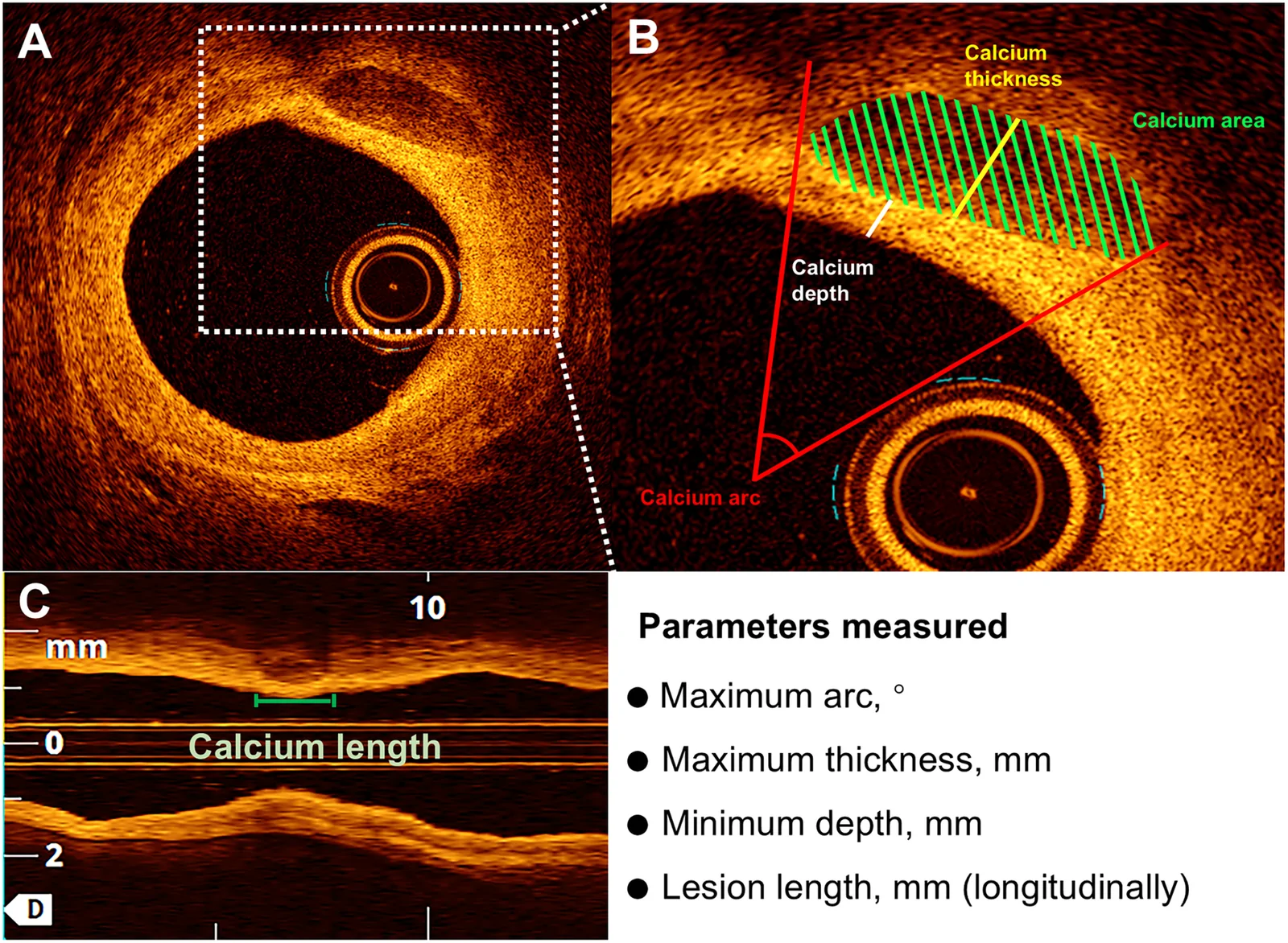
Quantitative OCT assessment of radial artery calcification lesions. **(A)** Cross-sectional OCT image of a calcified lesion. **(B)** Measurement of maximum calcium arc, maximum calcium thickness, and minimum calcium depth. **(C)** Longitudinal OCT view showing lesion-length measurement. Calcification area was not assessed because the complete abluminal calcium border was not consistently visible. OCT, optical coherence tomography; RAC, radial artery calcification.

An exploratory lesion-level RAC-OCT score (range, 0–10) was developed for this retrospective study, conceptually informed by coronary OCT calcium scoring and modified for radial artery morphology. The score combined calcium arc, thickness, length, and calcific nodule; component definitions and point assignments are provided in Supplementary Table S1. The cutoffs were investigator-defined during retrospective analysis and were neither optimized nor validated against clinical outcomes. No severity categories were applied.

### 2.5 Assessment of vessel-level radial artery plaque features

Prespecified vessel-level radial artery plaque features were recorded according to published radial and coronary OCT criteria (16, 17, 21–23). Fibrous plaque was identified by homogeneous, signal-rich intimal thickening; lipid plaque by a signal-poor region with diffuse borders and signal attenuation; microchannels by sharply delineated, signal-poor tubular or vesicular structures within the plaque without luminal communication; cholesterol crystals by thin, linear, high-intensity intraplaque structures; and macrophage accumulation by focal or confluent high-intensity punctate signals with signal attenuation. Each feature was recorded as present or absent for each radial artery and compared according to RAC status.

### 2.6 Coronary OCT analysis

An analyzable culprit-vessel coronary OCT examination was available for all 461 patients. The OCT-based calcium score was calculated for the culprit lesion according to Fujino et al. (24) (range, 0–4 points): 2 points for a maximum calcium arc >180°, 1 point for a maximum calcium thickness >0.5 mm, and 1 point for a calcium length >5 mm. The culprit-lesion score was used as the patient-level value, with one score per patient, and was not summed or maximized across multiple lesions. All imaged lesions within the culprit vessel were reviewed for calcific nodules. Patient-level status was positive when at least one OCT-defined calcific nodule was identified and negative when none was identified, irrespective of lesion or nodule count. Coronary OCT readers were blinded to the RA-OCT findings.

### 2.7 Reproducibility assessment

Patient-level RAC reproducibility was evaluated in a stratified random sample of 100 RA-OCT examinations, including 50 RAC-positive and 50 RAC-negative cases. Lesion-level reproducibility for morphologic pattern, depth pattern, macrophage accumulation, maximum calcium arc, maximum calcium thickness, and lesion length was evaluated in 50 randomly selected lesions from 50 patients. Because calcific nodules and cholesterol crystals were uncommon in the random sample, additional positive lesions were included in feature-specific analyses. Each patient contributed one lesion to each analysis, yielding 51 lesions for calcific nodule assessment (8 positive and 43 negative) and 52 lesions for cholesterol crystal assessment (6 positive and 46 negative). These enriched samples were used only for reproducibility assessment and not for prevalence estimation. Lesion length was used as the quantitative reproducibility measure of lesion-boundary placement.

Two independent readers, S.H. and J.Z., with 2 and 3 years of OCT interpretation experience, respectively, performed the assessments while blinded to clinical data, sampling stratum, reference classification, and each other's readings. S.H. repeated the assessment after 31 days using recoded and rerandomized images without access to the initial results. Interobserver disagreements in categorical classifications were adjudicated by J.G., who had 10 years of OCT interpretation experience. Reproducibility estimates were calculated from the original pre-adjudication readings.

### 2.8 Statistical analysis

Continuous variables are presented as mean ± SD or median [interquartile range], as appropriate, and categorical variables as *n* (%). Between-group comparisons used the Welch t test or Mann–Whitney U test for continuous variables and the chi-square test or Fisher exact test for categorical variables. Analyses were performed at the patient, segment, or lesion level, as appropriate. Proximal, middle, and distal RAC involvement was compared using Cochran's *Q* test, followed by two-sided exact McNemar tests with Holm correction. Segment-level morphometric and lesion-level OCT characteristics were summarized descriptively because multiple segments and lesions could arise from the same patient. Given the exploratory nature and heterogeneity of the analyses, no global multiplicity adjustment was applied. Unless otherwise specified, *P* values were two-sided and nominal, and effect estimates with 95% confidence intervals were emphasized. Baseline *P* values were not used for covariate selection.

The retrospective multivariable model was not formally preregistered. Covariates were selected before final model fitting according to clinical relevance, prior evidence, biological plausibility, and the study hypothesis; univariable *P*-value screening and automated stepwise selection were not used. Model 1 included age, sex, diabetes mellitus, renal insufficiency, prior ischemic stroke/transient ischemic attack, and reference radial artery diameter. Model 2 additionally included coronary calcium score and coronary calcific nodule. Glucose and HbA1c were not entered concurrently with diabetes mellitus, and albumin was not added solely because of an unadjusted group difference. Given the limited number of RAC-positive events, Model 2 was repeated using Firth penalized logistic regression. Peripheral artery disease, hypertension, and smoking were added individually in separate Firth sensitivity models, and eGFR replaced renal insufficiency in a separate model. All modeled variables had complete data, and no imputation was performed. Collinearity, linearity in the logit, and the functional form of coronary calcium score were assessed.

The five radial artery plaque features were evaluated in separate patient-level logistic regression models adjusted for age, sex, and diabetes mellitus. The Benjamini–Hochberg procedure was applied across the five comparisons, and Firth sensitivity models additionally adjusted for smoking history and peripheral artery disease. Reproducibility was assessed using unweighted Cohen's *κ* for categorical variables and absolute-agreement, single-measure intraclass correlation coefficients for continuous measurements; positive and negative agreement were additionally reported for patient-level RAC and selected lesion-level features. Ninety-five percent confidence intervals were obtained using 10,000 nonparametric bootstrap resamples at the patient or lesion level, as appropriate. Statistical analyses were performed using Microsoft Excel for Microsoft 365 and Python 3.13.5.

## 3 Results

### 3.1 Study population, RAC frequency, and baseline characteristics

Among 3,672 patients undergoing transradial PCI, 1,371 underwent OCT-guided PCI. Of these, 10 declined RA-OCT; RA-OCT was attempted in the remaining 1,361 patients (99.3%). After application of the imaging and access criteria, 461 patients constituted the final analytic cohort. The mean radial artery pullback length and analyzable length were 19.8 ± 1.7 cm and 19.2 ± 1.7 cm, respectively. OCT-defined RAC was identified in 61 patients (13.2%); 94 calcified segments and 214 calcified lesions were identified for segment- and lesion-level analyses. The complete selection process is shown in Figure 1.

Baseline characteristics are summarized descriptively in Table 1. Compared with RAC-negative patients, RAC-positive patients were older and more frequently had diabetes mellitus, prior ischemic stroke, and peripheral artery disease. They also had higher glucose and glycated hemoglobin levels and lower estimated glomerular filtration rate and albumin levels. These nominal comparisons were not used as an automatic screening criterion for multivariable modeling.

**Table 1:** Baseline characteristics according to radial artery calcification status.

Characteristic	RAC-negative (*n* = 400)	RAC-positive (*n* = 61)	*P* value
General characteristics			
Age, years	59.1 ± 12.6	72.0 ± 10.8	<0.001
Male sex	320 (80.0)	41 (67.2)	0.024
Body mass index, kg/m^2^	26.3 ± 3.3	26.3 ± 3.4	0.932
Reference radial artery diameter, mm	3.13 ± 0.29	3.12 ± 0.25	0.844
Cardiovascular risk factors			
Hypertension	248 (62.0)	45 (73.8)	0.075
Diabetes mellitus	130 (32.5)	36 (59.0)	<0.001
Dyslipidemia	335 (83.8)	48 (78.7)	0.326
Smoking history	235 (58.8)	30 (49.2)	0.159
Family history of CAD	86 (21.5)	8 (13.1)	0.130
Previous medical history			
Renal insufficiency	40 (10.0)	7 (11.5)	0.723
Dialysis	2 (0.5)	1 (1.6)	0.347
Prior stroke	37 (9.2)	17 (27.9)	<0.001
Ischemic stroke	34 (8.5)	15 (24.6)	<0.001
Peripheral artery disease	28 (7.0)	11 (18.0)	0.004
Previous medication			
Antiplatelet therapy	94 (23.5)	14 (23.0)	0.925
Statin therapy	117 (29.2)	13 (21.3)	0.199
Clinical diagnosis			
Unstable angina pectoris	148 (37.0)	22 (36.1)	0.990^a^
NSTEMI	78 (19.5)	12 (19.7)	
STEMI	174 (43.5)	27 (44.3)	
Laboratory findings			
Corrected calcium, mmol/L	2.19 ± 0.12	2.21 ± 0.11	0.099
Total cholesterol, mmol/L	4.46 ± 1.13	4.32 ± 1.36	0.454
HDL-C, mmol/L	1.10 ± 0.27	1.14 ± 0.29	0.270
LDL-C, mmol/L	2.85 ± 0.89	2.65 ± 1.10	0.171
Triglycerides, mmol/L	1.44 [1.05–2.13]	1.39 [0.93–2.07]	0.387
Lipoprotein(a), mg/L	138 [66–226]	129 [56–241]	0.556
Glucose, mmol/L	6.12 [5.17–7.63]	7.33 [6.19–9.17]	<0.001
HbA1c, %	6.0 [5.7–6.7]	6.4 [5.9–7.5]	0.008
Creatinine, μmol/L	78.0 [67.8–90.0]	78.0 [69.0–88.0]	0.904
eGFR, mL/min/1.73 m^2^	94.8 ± 31.6	73.6 ± 22.3	<0.001
Albumin, g/L	40.5 ± 3.4	38.8 ± 3.9	0.002
hs-CRP, mg/L	2.96 [1.04–9.70]	3.25 [1.35–13.96]	0.156

Values are mean ± SD, median [IQR], or *n* (%). Baseline characteristics are presented primarily for descriptive group characterization. Reported *P* values are two-sided and nominal, were not adjusted for multiplicity, and were not used as an automatic screening criterion for multivariable modeling. Tests were the Welch t test, Mann–Whitney U test, chi-square test, or Fisher exact test, as appropriate.

a The global *P* value for clinical diagnosis is shown on the first diagnostic category row.

CAD, coronary artery disease; eGFR, estimated glomerular filtration rate; HbA1c, glycated hemoglobin; HDL-C, high-density lipoprotein cholesterol; hs-CRP, high-sensitivity C-reactive protein; LDL-C, low-density lipoprotein cholesterol; NSTEMI, non-ST-segment elevation myocardial infarction; RAC, radial artery calcification; STEMI, ST-segment elevation myocardial infarction.

### 3.2 Anatomical distribution and extent of RAC

The anatomical distribution and extent of RAC are shown in Figures 5–7. Whole-vessel mapping provided a descriptive visualization of calcified lesion locations (Figure 5). All proximal, middle, and distal segments were evaluable in all 461 patients, yielding 1,383 assessed segments, of which 94 contained RAC (94/1,383, 6.8%). RAC involved the proximal, middle, and distal segments in 17/461 (3.7%), 25/461 (5.4%), and 52/461 (11.3%) patients, respectively (Figure 6). Among the 61 patients with RAC, these proportions corresponded to 17/61 (27.9%), 25/61 (41.0%), and 52/61 (85.2%), respectively; anatomical locations were not mutually exclusive.

**Figure 5:**
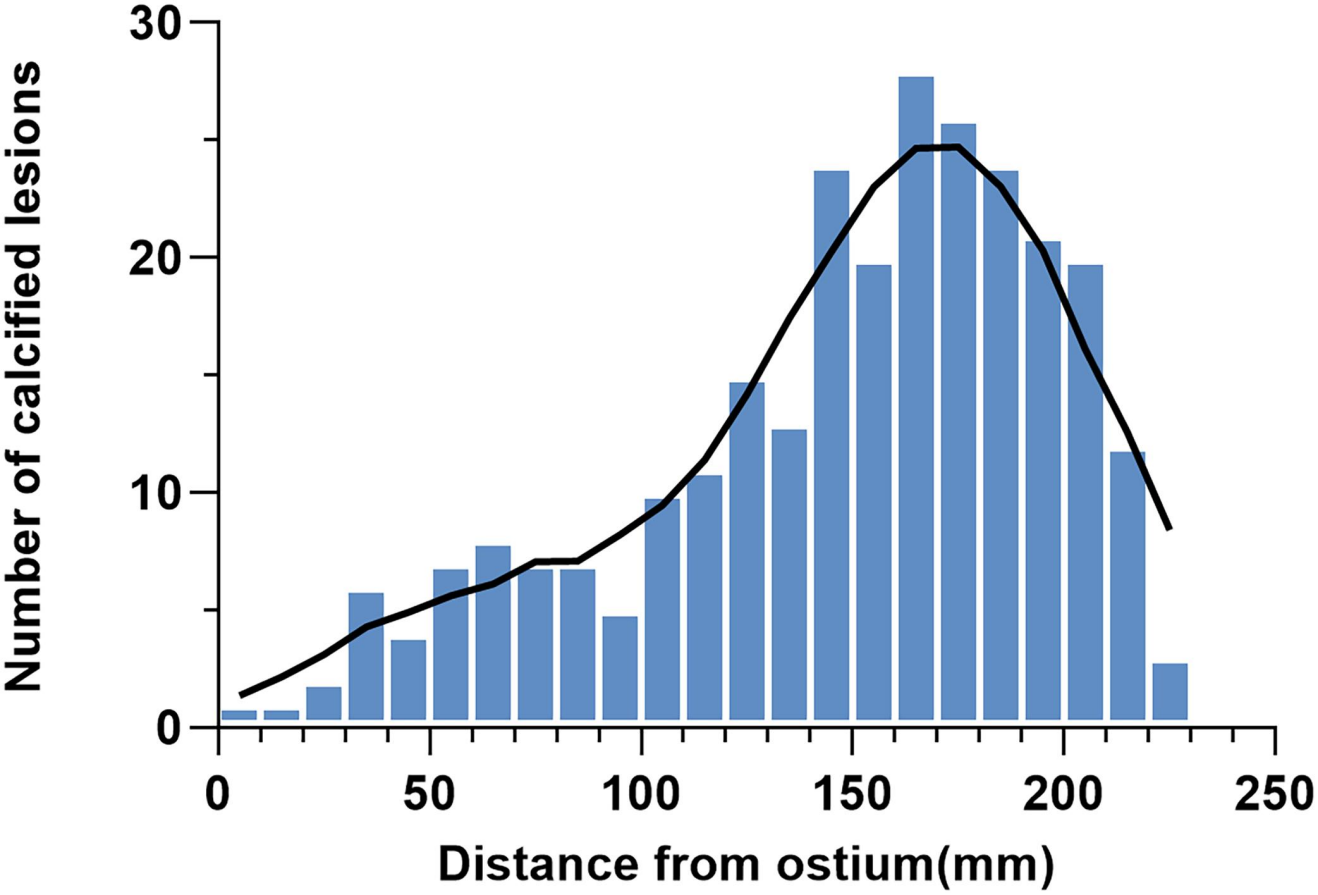
Whole-vessel spatial distribution of OCT-defined radial artery calcification. Calcified lesions are mapped according to their anatomical locations along the radial artery. Multiple lesions could arise from the same patient; therefore, this figure provides a descriptive visualization of lesion distribution rather than a segment-specific frequency estimate. OCT, optical coherence tomography; RAC, radial artery calcification.

**Figure 6:**
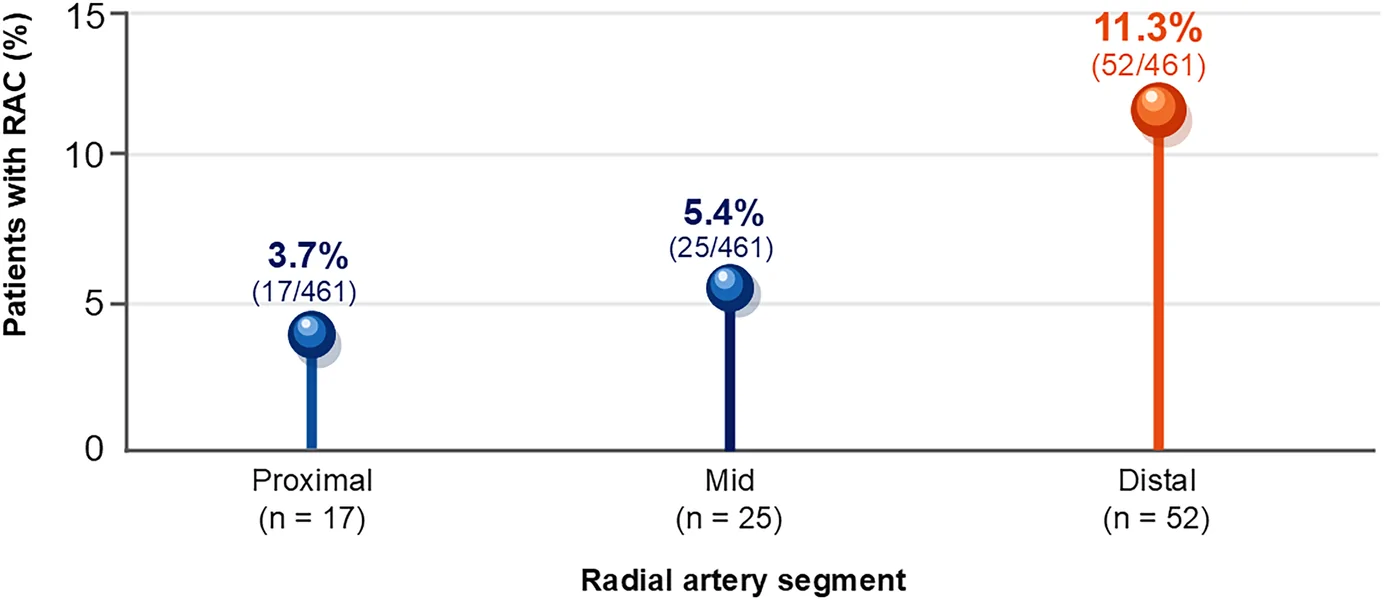
Frequency of OCT-defined radial artery calcification according to anatomical segment. Bars show patient-level frequencies, using 461 patients as the denominator for each location. Percentages are not mutually exclusive because more than one segment could be involved in the same patient. The overall comparison used Cochran's *Q* test, followed by two-sided exact McNemar tests with Holm adjustment for pairwise comparisons. OCT, optical coherence tomography; RAC, radial artery calcification.

**Figure 7:**
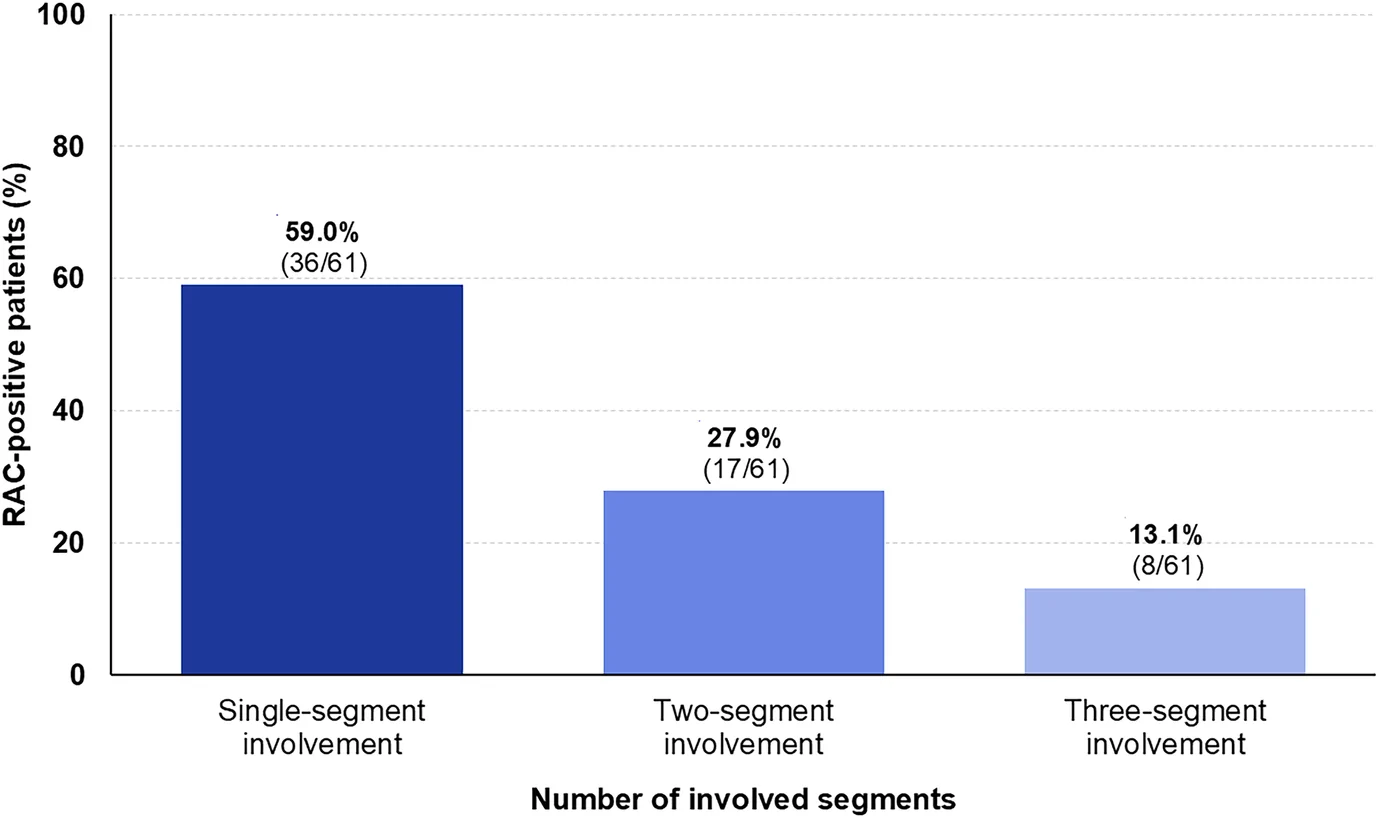
Extent of anatomical segment involvement among patients with RAC. Bars show the number and percentage of RAC-positive patients with one, two, or three involved segments; 61 RAC-positive patients constitute the denominator. RAC, radial artery calcification.

The frequency of segmental involvement differed across the three locations (Cochran's Q = 38.08, *P* < 0.001). Distal involvement was more frequent than middle and proximal involvement (both Holm-adjusted *P* < 0.001), whereas middle and proximal involvement did not differ significantly (Holm-adjusted *P* = 0.169). Among patients with RAC, 36/61 (59.0%) had single-segment involvement, 17/61 (27.9%) had two-segment involvement, and 8/61 (13.1%) had three-segment involvement (Figure 7). Segment-level morphometric and RAC-OCT score characteristics according to anatomical location are summarized descriptively in Supplementary Table S2.

### 3.3 Lesion-level OCT phenotypes and calcification burden

Lesion-level OCT phenotypes and calcification burden are shown in Figures 2, 3, 8. Among 214 calcified lesions, spotty calcification was the most common morphology (151/214, 70.6%), followed by sheet-like (35/214, 16.4%) and mixed-pattern calcification (28/214, 13.1%). Calcific nodules, assessed separately as an additional feature, were present in 8 lesions (3.7%). Superficial, deep/outer-wall, and mixed-depth calcification occurred in 110 (51.4%), 91 (42.5%), and 13 (6.1%) lesions, respectively. Median lesion length was 2.0 [1.0–3.4] mm, maximum calcium arc was 34.7 [21.9–57.6]°, maximum calcium thickness was 0.18 [0.10–0.28] mm, and minimum calcium depth was 0.09 [0.05–0.14] mm. The median lesion-level RAC-OCT score was 1.0 [0.0–2.0] (range, 0–10); its complete distribution is shown in Figure 8. These lesion-level findings are descriptive because multiple lesions could occur within the same patient. Detailed lesion-level characteristics are provided in Supplementary Table S3.

**Figure 8:**
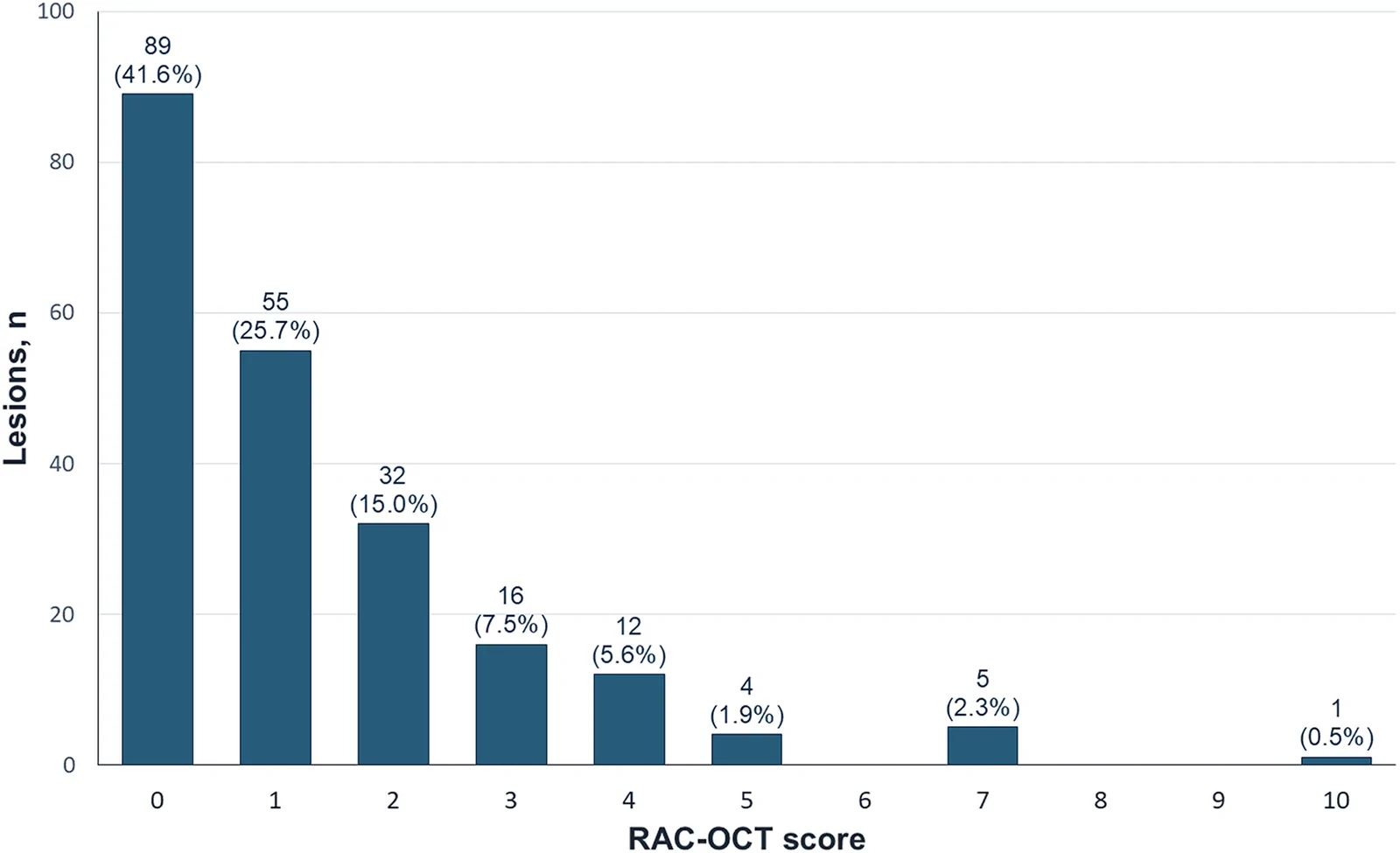
Distribution of lesion-level RAC-OCT scores. Bars show the number and percentage of lesions at each score. The exploratory score has not been validated against clinical outcomes. OCT, optical coherence tomography; RAC, radial artery calcification.

### 3.4 Vessel-level OCT plaque features according to RAC status

Unadjusted patient-level frequencies of fibrous plaque, lipid plaque, microchannels, cholesterol crystals, and macrophage accumulation were higher in RAC-positive than RAC-negative radial arteries (Supplementary Figure S1). In exploratory patient-level analyses, all five features were associated with RAC after adjustment for age, sex, and diabetes mellitus, and all associations remained significant after Benjamini–Hochberg false discovery rate correction. Findings were generally consistent in Firth sensitivity analyses, although residual confounding cannot be excluded. Detailed estimates and multiplicity-adjusted results are provided in Supplementary Table S4.

### 3.5 Coronary OCT variables and multivariable analyses

Coronary OCT was analyzable in all 461 patients, with no missing coronary calcium score or calcific nodule data. Coronary calcific nodules were present in 37 patients (8.0%). Detailed coronary procedural and culprit-vessel OCT characteristics according to RAC status are provided in Supplementary Table S5.

In Model 2, older age, diabetes mellitus, and coronary calcific nodule remained associated with RAC, whereas coronary calcium score did not retain statistical significance (Table 2). In the conventional model, the adjusted odds ratio for coronary calcific nodule was 7.21 (95% CI, 3.06–16.99); the Firth estimate was similar (adjusted OR, 6.64; 95% CI, 2.84–15.53).

**Table 2:** Factors associated with patient-level radial artery calcification.

Variable	Model 1: clinical model^a^		Model 2: clinical + imaging model^b^	
	OR (95% CI)	*P* value	OR (95% CI)	*P* value
Age, per 10 years	2.86 (2.06–3.98)	<0.001	2.16 (1.52–3.07)	<0.001
Male sex	1.19 (0.61–2.34)	0.613	1.12 (0.54–2.35)	0.755
Diabetes mellitus	2.86 (1.54–5.31)	<0.001	2.79 (1.43–5.42)	0.003
Renal insufficiency	0.68 (0.27–1.75)	0.424	0.92 (0.34–2.48)	0.864
Ischemic stroke/TIA	2.46 (1.16–5.22)	0.019	2.04 (0.88–4.69)	0.095
Reference radial artery diameter, per 0.1 mm	0.97 (0.86–1.08)	0.532	0.96 (0.85–1.08)	0.468
Coronary calcium score, per 1 point	—	—	1.19 (0.94–1.51)	0.140
Coronary calcific nodule	—	—	7.21 (3.06–16.99)	<0.001

CI, confidence interval; OR, odds ratio; RAC, radial artery calcification; TIA, transient ischemic attack.

a Odds ratios are shown with 95% confidence intervals. Model 1 included age, sex, diabetes mellitus, renal insufficiency, prior ischemic stroke/transient ischemic attack, and reference radial artery diameter.

b Model 2 included all variables in Model 1 and additionally included coronary calcium score and coronary calcific nodule. Coronary calcium score was modeled per one-point increase, whereas coronary calcific nodule was modeled as a binary variable. All 461 patients, including 61 with RAC, were included in both models; modeled variables had no missing data, and no imputation was performed.

All primary and sensitivity models included 461 patients, of whom 61 had RAC. Variance inflation factors ranged from 1.01 to 1.42, and the condition index was 1.98. There was no evidence of departure from linearity for age (*P* = 0.344) or reference radial artery diameter (*P* = 0.126). Categorical coding of coronary calcium score did not improve model fit over the per-one-point specification (likelihood-ratio *χ*^2^ = 1.70; 2 df; *P* = 0.427). The principal estimates remained consistent when peripheral artery disease, hypertension, or smoking was added individually and when eGFR replaced renal insufficiency. Detailed diagnostics and sensitivity results are provided in Supplementary Tables S6 and S7.

### 3.6 Reproducibility analyses

Patient-level RAC showed almost perfect interobserver and intraobserver agreement. Across lesion-level categorical variables, *κ* values ranged from 0.854 to 0.929 for interobserver agreement and from 0.852 to 0.925 for intraobserver agreement. Intraclass correlation coefficients for maximum calcium arc, maximum calcium thickness, and lesion length were ≥0.997. Detailed estimates are provided in Supplementary Table S8.

## 4 Discussion

Among the 461 analyzed patients, OCT-defined RAC was identified in 13.2%, occurred more frequently in the distal segment, and was usually confined to a single segment. Lesion-level phenotypes were heterogeneous and predominantly spotty. Exploratory patient-level analyses showed associations between RAC and multiple radial artery plaque features after adjustment and false discovery rate correction. Older age, diabetes mellitus, and coronary calcific nodule remained associated with RAC in the primary and sensitivity models, whereas coronary calcium score did not retain statistical significance.

### 4.1 Frequency and OCT-based assessment of RAC

The 13.2% frequency observed in our analytic cohort falls within the range reported in prior studies, although comparisons are limited by differences in study population, imaging modality, calcification definition, and sampling location. RAC has been reported in 8.6% of radial arteries assessed by intravascular ultrasound for coronary bypass conduit suitability (25) and in 51.0% of patients evaluated by Doppler ultrasound during distal radial access (15). Histopathological studies identified medial calcification in 6.3% of distal radial artery conduit specimens obtained during coronary artery bypass grafting (26), whereas studies in end-stage kidney disease or arteriovenous fistula cohorts reported RAC in 21.1%–36.7% of radial artery specimens or patients (27, 28). These estimates should not be interpreted as directly comparable population-level prevalence estimates.

Whole-vessel RA-OCT enabled assessment of RAC at the patient, segment, and lesion levels across a mean analyzable arterial length of 19.2 ± 1.7 cm. It provided *in vivo* information on longitudinal distribution, morphology, depth, and calcification burden that cannot be fully captured by focal ultrasound, angiography, computed tomography, or histological sampling.

### 4.2 Spatial distribution and lesion-level OCT phenotypes of RAC

In the present study, RAC involvement differed across the three paired anatomical locations and was more frequent distally. This pattern is consistent with previous radial artery OCT studies showing heterogeneous plaque distribution and distal clustering of calcified plaques (16, 17), and with ultrasound and radiographic data showing more distal upper-extremity arterial calcification in hemodialysis patients (29). The underlying mechanism remains unclear. Medial calcification, arterial stiffening, aging, and osteogenic differentiation of vascular smooth muscle cells may contribute to this distribution (29–33).

At the lesion level, RAC was classified using OCT descriptors adapted from coronary calcification assessment. Spotty morphology and superficial calcification were the most frequent patterns. The exploratory RAC-OCT score provided a descriptive 0–10 distribution but was not validated against procedural or clinical outcomes. Direct comparison with coronary calcification phenotypes remains inappropriate because of differences in vascular bed, lesion selection, and imaging indication.

### 4.3 RAC and coexisting radial artery atherosclerotic plaque features

In exploratory patient-level analyses, the associations between radial artery plaque features and RAC persisted after adjustment for age, sex, and diabetes mellitus and false discovery rate correction. This pattern may reflect a broader radial artery atherosclerotic phenotype beyond calcification alone. However, residual confounding remains possible, and the cross-sectional design does not establish temporal or causal relationships. The findings should therefore be considered hypothesis-generating.

### 4.4 Clinical and coronary calcification correlates of OCT-defined RAC

Evidence on factors associated with RAC remains limited and has largely been derived from ultrasound or histological studies in selected populations. Doppler ultrasound studies during distal radial access have linked RAC to older age, diabetes mellitus, chronic kidney disease, smoking, and coronary calcification variables (15). Histological studies in end-stage kidney disease and arteriovenous fistula cohorts have associated RAC with diabetes, dialysis duration, and mineral metabolism disorders (27, 28). Coronary calcific nodules are also recognized as a high-risk calcified plaque phenotype (34, 35). However, patient-level correlates of RAC assessed by whole-vessel radial artery OCT have not been reported.

In the present study, older age, diabetes mellitus, and coronary calcific nodule remained associated with OCT-defined RAC after adjustment, whereas coronary calcium score did not retain statistical significance. This should not be interpreted as evidence that one association was stronger than the other because calcific nodule was modeled as a binary variable, whereas calcium score was modeled per one-point increase. These cross-sectional findings identify correlates of RAC but do not establish prediction, temporality, or causality.

### 4.5 Clinical implications

RAC may be clinically relevant when the radial artery is considered for repeated transradial access, dialysis access, or coronary bypass conduit use. Previous studies have linked radial artery calcification to difficult or failed radial access (36–39). Severe radial artery calcification may complicate radiocephalic arteriovenous fistula construction or maturation, although successful maturation may still be achieved after careful ultrasonographic assessment (9, 40–42). Diffuse or extensive RAC is generally considered unfavorable for radial artery harvesting (26, 43, 44). These observations provide clinical context but should not be extrapolated directly to the present cohort.

The present study did not evaluate difficult access, radial spasm, catheter passage, access failure, radial artery occlusion or reuse, arteriovenous fistula outcomes, conduit suitability, or graft performance. Accordingly, OCT-defined RAC should be regarded as an imaging phenotype associated with clinical and coronary imaging characteristics, rather than a validated tool for clinical risk stratification. Its procedural and prognostic relevance requires prospective evaluation.

### 4.6 Limitations

This single-center retrospective observational study was restricted to patients with analyzable RA-OCT obtained after PCI during the same procedure, which may limit generalizability. Although RA-OCT was routinely attempted and was not prompted by suspected RAC, the observed 13.2% should not be interpreted as population prevalence. OCT does not provide histological confirmation, and depth-based categories remain imaging descriptors. The investigator-defined RAC-OCT score has not been validated against procedural or clinical outcomes. Procedural instrumentation during PCI via distal transradial access may have altered arterial wall morphology and affected the visualization or characterization of distal radial artery calcification. Without preprocedural RA-OCT for comparison, we cannot exclude an influence of instrumentation on the observed distal predominance of calcification, which should therefore be interpreted cautiously.

Model 2 included eight covariates but only 61 RAC-positive events (approximately 7.6 events per covariate), creating risks of model instability and overfitting. Although Firth and alternative-covariate sensitivity analyses yielded consistent estimates, small-sample bias and residual confounding remain possible. The cross-sectional design precludes conclusions regarding prediction, temporality, or causality.

Because multiple exploratory analyses were performed without global multiplicity correction, false-positive findings remain possible. Except where false discovery rate- or Holm-adjusted values are reported, *P* values are nominal, and secondary findings should be considered hypothesis-generating. These results require confirmation in larger independent cohorts.

## 5 Conclusions

In this cross-sectional study, OCT-defined RAC was identified in 13.2% of analyzed patients, was more frequent in the distal segment, and showed heterogeneous lesion-level phenotypes. Older age, diabetes mellitus, and coronary calcific nodule were associated with RAC; exploratory analyses also showed associations with multiple noncalcified radial artery plaque features. These findings require confirmation in independent cohorts.

## Data Availability

The original contributions presented in the study are included in the article/Supplementary Material, further inquiries can be directed to the corresponding author/s.
