# Supplementary material for: Optical coherence tomography–defined radial artery calcification: frequency, phenotypes, and correlates in a retrospective transradial PCI cohort
Source: Front Med (Lausanne). 2026 Oct 27;13:1945441. doi: 10.3389/fmed.2026.1945441 (PMC13748462; doi:10.3389/fmed.2026.1945441)
Supplement: Supplementary file 1 [file Supplementaryfile1.docx]

**Supplementary Material**

Optical Coherence Tomography–Defined Radial Artery Calcification: Frequency, Phenotypes, and Correlates in a Retrospective Transradial PCI Cohort

**Supplementary Figure S1. Comparison of OCT-defined radial artery plaque features between patients with and without RAC.**

Bars show unadjusted patient-level frequencies. RAC-positive, n = 61; RAC-negative, n = 400. Adjusted associations and multiplicity-corrected results are provided in Supplementary Table S4. OCT, optical coherence tomography; RAC, radial artery calcification.

**Supplementary Table S1. Components of the exploratory lesion-level RAC-OCT score.**

| **Score component** | **0 point** | **1 point** | **2 points** | **3 points** |
| --- | --- | --- | --- | --- |
| Arc score | <90° | 90–180° | 180–270° | ≥270° |
| Thickness score | <0.20 mm | 0.20–0.49 mm | ≥0.50 mm | — |
| Length score | <3 mm | 3–4.9 mm | 5–9.9 mm | ≥10 mm |
| Nodule score | Absent | Not applicable | Present | Not applicable |

The lesion-level RAC-OCT score is the sum of the arc, thickness, length, and nodule scores (range, 0–10 points). The score was constructed retrospectively for descriptive assessment of calcification burden. Component cutoffs were investigator-defined and were neither optimized nor validated against clinical outcomes. No severity categories were applied. OCT, optical coherence tomography; RAC, radial artery calcification.

**Supplementary Table S2. Segment-level RAC characteristics by anatomical location among patients with RAC.**

| **Characteristic** | **Overall** | **Proximal** | **Middle** | **Distal** |
| --- | --- | --- | --- | --- |
| Segments with RAC among RAC-positive patients, n/N (%) | 94/183 (51.4) | 17/61 (27.9) | 25/61 (41.0) | 52/61 (85.2) |
| Calcified lesions, n | 214 | 26 | 59 | 129 |
| Total calcification length per calcified segment, mm | 3.5 [1.7, 11.0] | 2.6 [1.4, 4.6] | 3.8 [2.0, 12.0] | 4.9 [1.8, 13.4] |
| Average maximum calcium arc, ° | 36.2 [28.3, 61.9] | 36.6 [15.3, 62.5] | 33.6 [30.0, 52.0] | 40.9 [29.0, 66.7] |
| Average maximum calcium thickness, mm | 0.22 [0.13, 0.37] | 0.15 [0.08, 0.29] | 0.19 [0.16, 0.39] | 0.23 [0.13, 0.38] |
| Average minimum calcium depth, mm | 0.11 [0.06, 0.17] | 0.12 [0.08, 0.17] | 0.12 [0.04, 0.19] | 0.10 [0.05, 0.15] |
| Calcified lesions per calcified segment | 2 [1, 3] | 1 [1, 2] | 2 [1, 3] | 2 [1, 3] |
| Segment-level maximum RAC-OCT score | 2 [1, 3] | 1 [0, 2] | 2 [1, 3] | 2 [1, 3] |
| Segment-level cumulative RAC-OCT score | 2 [1, 4] | 1 [0, 2] | 2 [1, 3] | 2 [1, 6] |
| Radial artery diameter, mm | 3.06 [2.79, 3.58] | 3.30 [3.00, 3.85] | 3.05 [2.85, 3.57] | 2.85 [2.62, 3.26] |

Values are median [interquartile range], n/N (%), or counts. In the overall cohort, 94 of 1,383 assessed segments (6.8%) contained RAC; location-specific prevalence was 17/461 (3.7%) for proximal, 25/461 (5.4%) for middle, and 52/461 (11.3%) for distal segments. The table otherwise summarizes segment-level characteristics among the 61 RAC-positive patients, in whom 183 anatomical segments were evaluated and 94 contained RAC. Segment-level morphometric and score variables were summarized among the 94 calcified segments; radial artery diameter was summarized across all 183 segments. Because multiple calcified segments could arise from the same patient, morphometric and score comparisons are descriptive and no independence-based P values are shown. The patient-level comparison of segment involvement used Cochran’s Q test (Q = 38.08; P < 0.001), followed by exact McNemar tests with Holm adjustment: distal versus middle and distal versus proximal, both adjusted P < 0.001; middle versus proximal, adjusted P = 0.169. OCT, optical coherence tomography; RAC, radial artery calcification.

**Supplementary Table S3. Lesion-level OCT characteristics by anatomical location.**

| **Characteristic** | **Overall** | **Proximal** | **Middle** | **Distal** |
| --- | --- | --- | --- | --- |
| Calcified lesions, n | 214 | 26 | 59 | 129 |
| Lesion length, mm | 2.0 [1.0, 3.4] | 2.0 [1.0, 3.0] | 1.8 [0.8, 3.1] | 2.0 [1.0, 3.6] |
| Maximum calcium arc, ° | 34.7 [21.9, 57.6] | 25.9 [13.9, 61.2] | 34.2 [25.1, 52.2] | 36.5 [22.7, 59.5] |
| Maximum calcium thickness, mm | 0.18 [0.10, 0.28] | 0.19 [0.08, 0.28] | 0.17 [0.11, 0.27] | 0.18 [0.10, 0.32] |
| Minimum calcium depth, mm | 0.09 [0.05, 0.14] | 0.11 [0.08, 0.15] | 0.10 [0.05, 0.15] | 0.08 [0.05, 0.14] |
| Lesion-level RAC-OCT score | 1 [0, 2] | 1 [0, 2] | 1 [0, 2] | 1 [0, 2] |
| Superficial calcification | 110 (51.4) | 10 (38.5) | 29 (49.2) | 71 (55.0) |
| Deep/outer-wall calcification | 91 (42.5) | 14 (53.8) | 28 (47.5) | 49 (38.0) |
| Mixed-depth calcification | 13 (6.1) | 2 (7.7) | 2 (3.4) | 9 (7.0) |
| Spotty morphology | 151 (70.6) | 20 (76.9) | 44 (74.6) | 87 (67.4) |
| Sheet-like morphology | 35 (16.4) | 2 (7.7) | 7 (11.9) | 26 (20.2) |
| Mixed-pattern morphology | 28 (13.1) | 4 (15.4) | 8 (13.6) | 16 (12.4) |
| Calcific nodule | 8 (3.7) | 1 (3.8) | 0 (0.0) | 7 (5.4) |

Values are median [interquartile range], n (%), or counts. Spotty, sheet-like, and mixed-pattern calcification were mutually exclusive morphologic patterns; calcific nodule was assessed separately as a non-mutually exclusive feature. The 214 lesions were clustered within 61 RAC-positive patients. Lesion-level results are descriptive; lesions were not treated as independent observations and no independence-based P values are shown. The RAC-OCT score is presented as a continuous descriptive measure, and no severity categories were applied. OCT, optical coherence tomography; RAC, radial artery calcification.

**Supplementary Table S4. Exploratory patient-level associations between radial artery plaque features and OCT-defined RAC.**

| **Plaque feature** | **RAC-positive n/N (%)** | **RAC-negative n/N (%)** | **Adjusted OR (95% CI)ᵃ** | **Nominal P value** | **FDR-adjusted q value** | **Firth sensitivity OR (95% CI)ᵇ** |
| --- | --- | --- | --- | --- | --- | --- |
| Fibrous plaque | 34/61 (55.7) | 42/400 (10.5) | 8.85 (4.50–17.39) | <0.001 | <0.001 | 8.60 (4.35–17.01) |
| Lipid plaque | 41/61 (67.2) | 32/400 (8.0) | 17.09 (8.37–34.88) | <0.001 | <0.001 | 15.44 (7.58–31.45) |
| Macrophage accumulation | 18/61 (29.5) | 13/400 (3.2) | 6.10 (2.60–14.33) | <0.001 | <0.001 | 5.42 (2.29–12.83) |
| Cholesterol crystals | 6/61 (9.8) | 8/400 (2.0) | 6.32 (1.65–24.25) | 0.007 | 0.007 | 5.66 (1.42–22.56) |
| Microchannels | 28/61 (45.9) | 73/400 (18.2) | 3.04 (1.59–5.81) | <0.001 | <0.001 | 2.91 (1.53–5.53) |

ᵃ Each plaque feature was entered in a separate logistic regression model adjusted for age, sex, and diabetes mellitus. Benjamini–Hochberg false discovery rate correction was applied across the five comparisons; q values are the resulting FDR-adjusted P values. ᵇ Sensitivity estimates were obtained using Firth penalized logistic regression adjusted for age, sex, diabetes mellitus, smoking history, and peripheral artery disease; confidence intervals are Wald-type intervals. Absence of the corresponding plaque feature was the reference category. All 461 patients had complete data for the modeled variables. These analyses were exploratory and hypothesis-generating. CI, confidence interval; FDR, false discovery rate; OCT, optical coherence tomography; OR, odds ratio; RAC, radial artery calcification.

**Supplementary Table S5. Coronary procedural and culprit-vessel OCT characteristics according to RAC status.**

| **Characteristic** | **Overall (N=461)** | **RAC-negative (n=400)** | **RAC-positive (n=61)** | **P value** |
| --- | --- | --- | --- | --- |
| **Panel A. Coronary procedural characteristics** | | | | |
| Culprit vessel: LAD | 255 (55.3) | 220 (55.0) | 35 (57.4) | 0.235 |
| Culprit vessel: LCX | 64 (13.9) | 60 (15.0) | 4 (6.6) |  |
| Culprit vessel: RCA | 139 (30.2) | 118 (29.5) | 21 (34.4) |  |
| Culprit vessel: LM | 3 (0.7) | 2 (0.5) | 1 (1.6) |  |
| Number of diseased vessels | 2 [1, 3] | 2 [1, 3] | 3 [2, 3] | <0.001 |
| Multivessel disease (≥2 vessels) | 337 (73.1) | 282 (70.5) | 55 (90.2) | 0.002 |
| PCI performed | 421 (91.3) | 365 (91.2) | 56 (91.8) | 1.000 |
| Intravascular lithotripsy | 15 (3.3) | 14 (3.5) | 1 (1.6) | 0.705 |
| Orbital atherectomy | 8 (1.7) | 5 (1.2) | 3 (4.9) | 0.076 |
| Number of coronary OCT-imaged vessels | 2 [2, 3] | 3 [2, 3] | 2 [1, 3] | 0.002 |
| **Panel B. Culprit-vessel coronary OCT characteristics** | | | | |
| Coronary calcification | 331 (71.8) | 276 (69.0) | 55 (90.2) | 0.001 |
| Maximum calcium arc, ° | 122.6 [78.8, 196.9] | 115.1 [73.1, 172.7] | 197.3 [125.6, 266.0] | <0.001 |
| Maximum calcium thickness, mm | 0.93 [0.69, 1.19] | 0.91 [0.68, 1.18] | 1.01 [0.86, 1.35] | 0.006 |
| Coronary calcium score | 2 [0, 2] | 1 [0, 2] | 4 [2, 4] | <0.001 |
| Coronary calcific nodule | 37 (8.0) | 14 (3.5) | 23 (37.7) | <0.001 |
| Eruptive calcific nodule | 6 (1.3) | 2 (0.5) | 4 (6.6) |  |
| Non-eruptive calcific nodule | 31 (6.7) | 12 (3.0) | 19 (31.1) |  |
| Fibrous plaque | 417 (90.5) | 365 (91.2) | 52 (85.2) | 0.210 |
| Thin-cap fibroatheroma | 55 (11.9) | 43 (10.8) | 12 (19.7) | 0.073 |
| Lipid plaque | 411 (89.2) | 354 (88.5) | 57 (93.4) | 0.375 |
| Fibrous cap thickness, mm | 0.05 [0.05, 0.06] | 0.05 [0.05, 0.06] | 0.06 [0.05, 0.06] | 0.582 |
| Maximum lipid arc, ° | 174.9 [131.2, 258.0] | 175.1 [129.9, 262.3] | 167.6 [132.1, 220.7] | 0.404 |
| Plaque rupture | 174 (37.7) | 150 (37.5) | 24 (39.3) | 0.893 |
| Plaque erosion | 107 (23.2) | 99 (24.8) | 8 (13.1) | 0.065 |
| Thrombus | 175 (38.0) | 156 (39.0) | 19 (31.1) | 0.300 |
| Microchannel | 134 (29.1) | 125 (31.2) | 9 (14.8) | 0.013 |
| Macrophage accumulation | 160 (34.7) | 129 (32.2) | 31 (50.8) | 0.007 |
| Cholesterol crystals | 128 (27.8) | 102 (25.5) | 26 (42.6) | 0.009 |
| Minimum lumen area, mm² | 1.42 [1.02, 2.17] | 1.38 [1.02, 2.16] | 1.60 [1.05, 2.28] | 0.334 |

Values are median [interquartile range] or n (%). For multi-category variables, one overall P value is shown in the first category row. Calcium arc and thickness were summarized among patients with coronary calcification; fibrous cap thickness among patients with nonzero measured cap thickness; and maximum lipid arc among patients with lipid plaque. LAD, left anterior descending artery; LCX, left circumflex artery; LM, left main artery; OCT, optical coherence tomography; PCI, percutaneous coronary intervention; RAC, radial artery calcification; RCA, right coronary artery. P values are two-sided, nominal, unadjusted for multiplicity, and descriptive.

**Supplementary Table S6. Model sample sizes, collinearity diagnostics, and functional-form assessments.**

| **Model or assessment** | **N / RAC events** | **Missing, n** | **Result or specification** |
| --- | --- | --- | --- |
| **Panel A. Analysis sets and missingness** | | | |
| Conventional Model 1 | 461 / 61 | 0 | Complete-case analysis; no imputation |
| Conventional Model 2 | 461 / 61 | 0 | Eight covariates; approximately 7.6 events per covariate |
| Primary Firth Model 2 | 461 / 61 | 0 | Same covariates as conventional Model 2 |
| Firth Model 2 + peripheral artery disease | 461 / 61 | 0 | Peripheral artery disease added individually |
| Firth Model 2 + hypertension | 461 / 61 | 0 | Hypertension added individually |
| Firth Model 2 + smoking history | 461 / 61 | 0 | Smoking history added individually |
| Firth Model 2 with eGFR replacing renal insufficiency | 461 / 61 | 0 | eGFR replaced renal insufficiency; no missing data |
| **Panel B. Diagnostic or functional-form assessment** | | | |
| Variance inflation factors, Model 2 | 461 / 61 | — | Range, 1.01–1.42 |
| Condition index, Model 2 | 461 / 61 | — | 1.98 |
| Age: linearity in the logit | 461 / 61 | — | Box–Tidwell-type likelihood-ratio test, P=0.344 |
| Reference radial artery diameter: linearity in the logit | 461 / 61 | — | Box–Tidwell-type likelihood-ratio test, P=0.126 |
| Coronary calcium score: per-point vs categorical coding | 461 / 61 | — | Likelihood-ratio χ²=1.70; 2 df; P=0.427 |

Model 1 included age, sex, diabetes mellitus, renal insufficiency, prior ischemic stroke/transient ischemic attack, and reference radial artery diameter. Model 2 additionally included coronary calcium score and coronary calcific nodule. The categorical coronary calcium score specification used 0, 1, 2, and ≥3 points. eGFR, estimated glomerular filtration rate; RAC, radial artery calcification.

**Supplementary Table S7. Firth penalized and alternative-covariate sensitivity analyses for patient-level RAC.**

**Panel A. Full primary Firth Model 2**

| **Variable** | **Adjusted OR (95% CI)** | **P value** |
| --- | --- | --- |
| Age, per 1 year | 1.08 (1.04–1.11) | <0.001 |
| Male sex | 1.11 (0.54–2.28) | 0.781 |
| Diabetes mellitus | 2.69 (1.41–5.15) | 0.003 |
| Renal insufficiency | 0.96 (0.37–2.52) | 0.935 |
| Prior ischemic stroke/TIA | 2.02 (0.89–4.57) | 0.093 |
| Reference radial artery diameter, per 1 mm | 0.67 (0.22–2.08) | 0.489 |
| Coronary calcium score, per 1 point | 1.19 (0.94–1.49) | 0.143 |
| Coronary calcific nodule | 6.64 (2.84–15.53) | <0.001 |

**Panel B. Alternative-covariate Firth sensitivity analyses**

| **Sensitivity model** | **N / events** | **Coronary calcific nodule** | **Coronary calcium score** | **Added/substituted covariate** |
| --- | --- | --- | --- | --- |
| Primary Firth Model 2 | 461 / 61 | 6.64 (2.84–15.53); P<0.001 | 1.19 (0.94–1.49); P=0.143 | Not applicable |
| + Peripheral artery disease | 461 / 61 | 6.43 (2.75–15.01); P<0.001 | 1.20 (0.95–1.51); P=0.122 | 1.98 (0.82–4.76); P=0.127 |
| + Hypertension | 461 / 61 | 6.59 (2.82–15.39); P<0.001 | 1.19 (0.94–1.49); P=0.146 | 1.04 (0.51–2.12); P=0.913 |
| + Smoking history | 461 / 61 | 6.47 (2.75–15.21); P<0.001 | 1.19 (0.95–1.50); P=0.137 | 1.12 (0.52–2.41); P=0.763 |
| Replace renal insufficiency with eGFR | 461 / 61 | 6.94 (2.94–16.40); P<0.001 | 1.19 (0.94–1.49); P=0.143 | 0.96 (0.82–1.13); P=0.660 |

All primary and sensitivity models included 461 patients (61 RAC-positive events), and the modeled variables had no missing data; no imputation was performed. In Panel B, peripheral artery disease, hypertension, and smoking history were added individually to Firth Model 2; eGFR replaced renal insufficiency because both represent renal function. eGFR was modeled per 10 mL/min/1.73 m² increase. These exploratory analyses assess consistency but do not eliminate the limitations imposed by 61 RAC-positive events. CI, confidence interval; eGFR, estimated glomerular filtration rate; OR, odds ratio; RAC, radial artery calcification; TIA, transient ischemic attack.

**Supplementary Table S8. Interobserver and intraobserver reproducibility of OCT-defined radial artery calcification assessments.**

| **Variable** | **Analysis set** | **Statistic** | **Interobserver estimate (95% CI)** | **Intraobserver estimate (95% CI)** | Overall agreement, % (inter/intra) |
| --- | --- | --- | --- | --- | --- |
| Patient-level RAC | 100 examinations | κ | 0.940 (0.860–1.000) | 0.960 (0.899–1.000) | 97.0 / 98.0 |
| Morphologic pattern | 50 lesions | κ | 0.920 (0.788–1.000) | 0.915 (0.782–1.000) | 96.0 / 96.0 |
| Depth pattern | 50 lesions | κ | 0.923 (0.805–1.000) | 0.925 (0.815–1.000) | 96.0 / 96.0 |
| Calcific nodule | 51 lesions | κ | 0.929 (0.738–1.000) | 0.852 (0.580–1.000) | 98.0 / 96.1 |
| Macrophage accumulation | 50 lesions | κ | 0.854 (0.660–1.000) | 0.901 (0.733–1.000) | 94.0 / 96.0 |
| Cholesterol crystals | 52 lesions | κ | 0.898 (0.639–1.000) | 0.912 (0.649–1.000) | 98.1 / 98.1 |
| Maximum calcium arc, ° | 50 lesions | ICC | 0.9998 (0.9995–0.9999) | 0.9999 (0.9998–1.0000) | — |
| Maximum calcium thickness, mm | 50 lesions | ICC | 0.9973 (0.9939–0.9991) | 0.9990 (0.9970–0.9997) | — |
| Lesion length, mm | 50 lesions | ICC | 0.9998 (0.9963–0.9999) | 0.9999 (0.9986–1.0000) | — |

Interobserver agreement compares S.H.’s first assessment with J.Z.’s assessment; intraobserver agreement compares S.H.’s first and second assessments after 31 days. Positive/negative agreement for interobserver and intraobserver assessments, respectively, was 97.0%/97.0% and 98.0%/98.0% for patient-level RAC; 94.1%/98.8% and 87.5%/97.7% for calcific nodule; 89.7%/95.8% and 92.9%/97.2% for macrophage accumulation; and 90.9%/98.9% and 92.3%/98.9% for cholesterol crystals. Calcific nodule and cholesterol crystal analyses used feature-enriched samples with one lesion per patient and were not used to estimate prevalence. Reproducibility estimates were calculated from the original pre-adjudication readings. CIs were obtained using 10,000 nonparametric bootstrap resamples. CI, confidence interval; ICC, absolute-agreement, single-measure intraclass correlation coefficient; κ, Cohen’s kappa coefficient; OCT, optical coherence tomography; RAC, radial artery calcification.
